# Sequence and Ionomic Analysis of Divergent Strains of Maize Inbred Line B73 with an Altered Growth Phenotype

**DOI:** 10.1371/journal.pone.0096782

**Published:** 2014-05-07

**Authors:** Martin Mascher, Nina Gerlach, Manfred Gahrtz, Marcel Bucher, Uwe Scholz, Thomas Dresselhaus

**Affiliations:** 1 Department of Cytogenetics and Genome Analysis, Leibniz Institute of Plant Genetics and Crop Plant Research (IPK), Corrensstraβe 3, Stadt Seeland, Germany; 2 Botanical Institute, Cologne Biocenter, Cluster of Excellence on Plant Sciences (CEPLAS), University of Cologne, Zülpicherstrasse 47b, Cologne, Germany; 3 Cell Biology and Plant Biochemistry, Biochemie-Zentrum Regensburg, University of Regensburg, Universitätsstraβe 31, Regensburg, Germany; Universidad Miguel Hernández de Elche, Spain

## Abstract

Maize (*Zea mays*) is the most widely grown crop species in the world and a classical model organism for plant research. The completion of a high-quality reference genome sequence and the advent of high-throughput sequencing have greatly empowered re-sequencing studies in maize. In this study, plants of maize inbred line B73 descended from two different sets of seed material grown for several generations either in the field or in the greenhouse were found to show a different growth phenotype and ionome under phosphate starvation conditions and moreover a different responsiveness towards mycorrhizal fungi of the species *Glomus intraradices* (syn: *Rhizophagus irregularis*). Whole genome re-sequencing of individuals from both sets and comparison to the B73 reference sequence revealed three cryptic introgressions on chromosomes 1, 5 and 10 in the line grown in the greenhouse summing up to a total of 5,257 single-nucleotide polymorphisms (SNPs). Transcriptome sequencing of three individuals from each set lent further support to the location of the introgression intervals and confirmed them to be fixed in all sequenced individuals. Moreover, we identified >120 genes differentially expressed between the two B73 lines. We thus have found a nearly-isogenic line (NIL) of maize inbred line B73 that is characterized by an altered growth phenotype under phosphate starvation conditions and an improved responsiveness towards symbiosis with mycorrhizal fungi. Through next-generation sequencing of the genomes and transcriptomes we were able to delineate exact introgression intervals. Putative *de novo* mutations appeared approximately uniformly distributed along the ten maize chromosomes mainly representing G:C -> A:T transitions. The plant material described in this study will be a valuable tool both for functional studies of genes differentially expressed in both B73 lines and for research on growth behavior especially in response to symbiosis between maize and mycorrhizal fungi.

## Introduction

Maize (Z. *mays*) is an important cereal crop and has been a major plant model species for genetic research since the first half of the 20^th^ century. It is an extremely diverse species whose genome abounds with single-nucleotide polymorphisms (SNPs) [1], [2] as well as with copy number and presence-absence variation between inbred lines [2], [3]. Dedicated stock centers maintain phenotypically characterized mutant lines as well as cultivars of former and present agricultural importance. A community-driven online database [4] provides a searchable catalogue of morphological and cytological variation captured in elite cultivars, landraces as well as wild accessions and facilitates the distribution of seed material to researchers around the world.

Like its wild progenitor teosinte, maize is a predominantly outcrossing crop. However, artificial self-pollination is possible and commonly used in breeding programs. Inbred lines, i.e. nearly homozygous individuals, can be easily generated and maintained by repeated self-fertilization. One of the most widely used inbred lines of maize is named as B73. Developed at Iowa State University, B73 is among the founder lines of the so-called stiff-stalk germplasm group. Consequently, a recent re-sequencing study found more than 50 inbred lines from different breeding programs to be closely related to B73 [5]. In a scientific context, B73 is one parent of the IBM intermated recombinant inbred line (RIL) mapping population [6] and is the common parent shared by all lines of the maize nested association mapping population [7]. The genome of B73 had been sequenced to high quality using a map-based clone-by-clone strategy [8]. This sequence resource now represents an invaluable resource for re-sequencing studies. Haplotype maps (HapMaps) that include hundred of thousands to millions of variant positions genotyped in hundreds to tens of thousands of individuals have been constructed by whole-genome or reduced representation sequencing [1], [2], [5]. The B73 reference sequence thus provides the backbone for genome-wide association studies [9] and map-based cloning projects [10] for the crop and model plant maize.

Maize like most other terrestrial plants can form symbiotic relationships with arbuscular mycorrhizal (AM) fungi. These soil-born fungi are obligate symbionts that colonize plant roots and assist their hosts in the uptake of water and nutrients, in particular phosphate (Pi), while obtaining plant carbohydrates in exchange (see [11] for a review). Transporters specific for nutrients like P, S or Zn or plant sugars functioning at the plant/fungus interface mediate mycorrhiza-specific exchange processes [12]–[14]. The physiological response of plants to AM symbiosis is variable and strongly depends on environmental conditions and the genotypic background [15]–[17]. Even between replicate experiments, mycorrhizal responsiveness has been described to vary depending on growth seasons [18]. In maize, Kaeppler et al. [19] evaluated the responsiveness of different maize lines including B73 towards mycorrhizal colonization. In this study, line B73 robustly showed a strong increase in biomass generation under low Pi condition in the presence of AM fungi. Moreover, one QTL controlling mycorrhiza responsiveness was found on chromosome #2 whereas three QTLs for Pi starvation response in absence of mycorrhiza were located on other chromosomal regions [19].

Here, we describe an inbred line of maize that is nearly isogenic to B73 used to generate the reference genome. This line was propagated for more than 20 years exclusively in the greenhouse without intended exposure to the mycorrhizal fungus *G. intraradices*. It phenotypically differs significantly from B73 in its responsiveness towards symbiosis with AM under limited availability of Pi. Additionally, it shows a different growth phenotype and ion composition under low Pi conditions in absence of mycorrhizal fungi. Through whole genome and transcriptome comparison with the B73 reference genome and a set of B73 plants that were obtained more recently from the stock center and exclusively grown in the field, we show that the genome of this nearly isogenic line (NIL) harbors three well-delineated segments from a different maize genotype in a B73 background summing up to a total of 5,257 SNPs. *De novo* mutation were found throughout the genome mostly representing G:C -> A:T transitions. Finally, we report and discuss >120 genes differently expressed between the two B73 strains potentially associated with the above described growth phenotypes.

## Results

In the course of a project that involved the collection of transcriptome, ionome and metabolome data from a large number of maize inbred lines [20], we grew, amongst others, plants of maize inbred line B73 under various growth conditions. B73 seeds had been obtained from two different sources. One set of seeds (set A) is descended from seed material obtained from the USDA stock center in 2007 (Acc.-No.: PI 550473) and was further propagated after self-pollination for three generations in the field. The second set of seeds (set B) had been obtained as B73 seeds from the USDA stock center in the early 1990s. Plants descended from this seed material were propagated after self-pollination for more than 20 generations exclusively in green houses at the universities of Hamburg and Regensburg, respectively.

### Growth Response towards Phosphate Starvation and Arbuscular Mycorrhizal Colonisation

In the course of our experiments, plants from both sets together with other inbred lines were grown in a bicompartmented system including or lacking arbuscular mycorrhizal fungi (+AM, −AM). One compartment was supplemented with Pi and closed by a hyphae-permeable membrane (hyphal compartment, HC) as already described [21]. By using this method, fungal hyphae link the Pi source with the maize root system and therefore enable Pi uptake via the mycorrhizal Pi uptake pathway. Under these conditions, −AM plants highly suffer from Pi deficiency, while +AM were supplied by Pi out of the HC [21].

**Figure 1:**
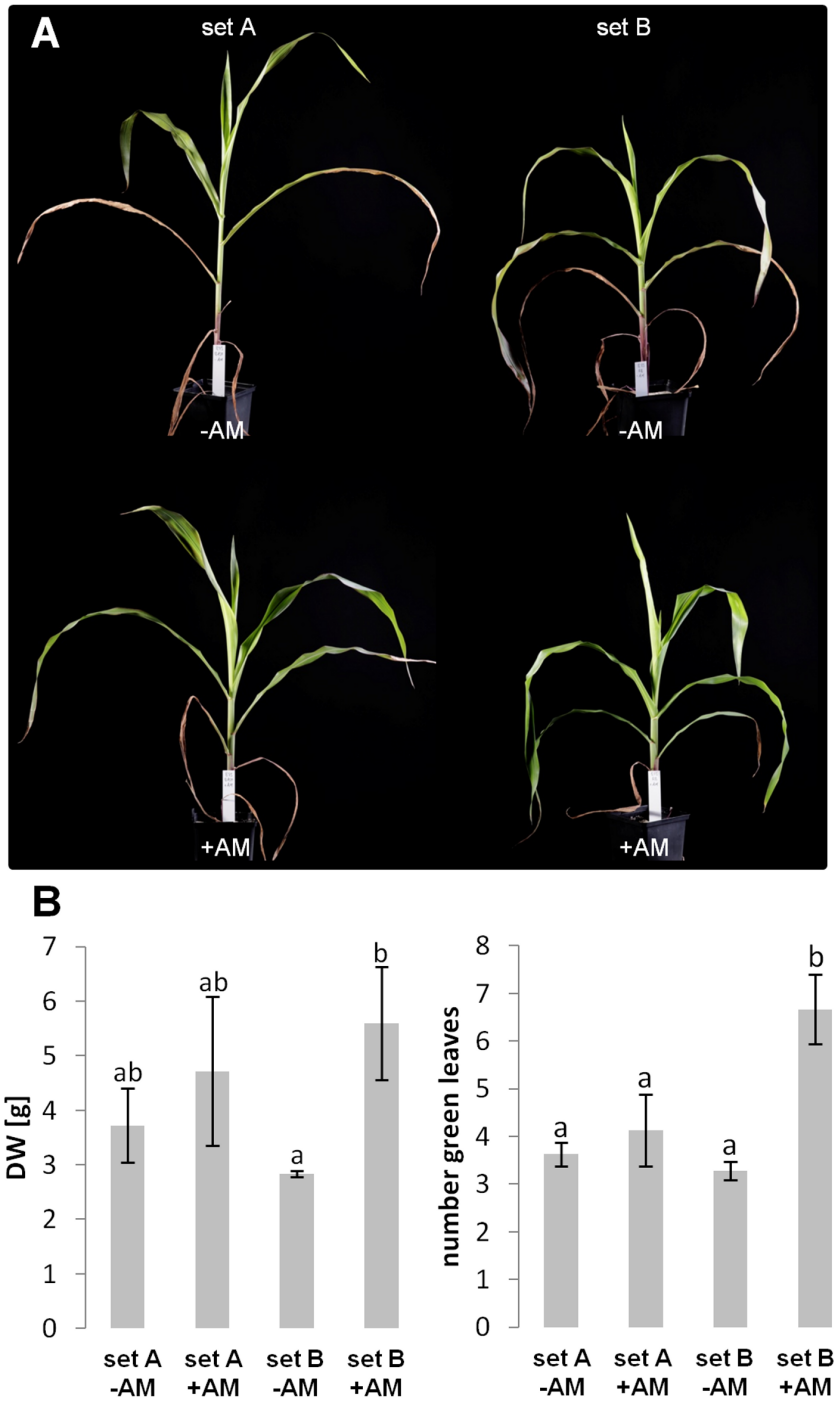
Phenotypic comparison of progeny plants from two maize B73 inbred lines grown for generations either exclusively in the field (set A) or in the greenhouse (set B). Plants were grown in compartmented pots with (+AM) or without (−AM) arbuscular mycorrhiza with phosphate addition to the hyphal compartment. (**A**) Comparison of plant growth phenotype. Pictures have been taken before harvest, approx. 7 weeks after sowing. (**B**) Comparison of dry weight (DW) and number of green leaves from 3–4 pooled plants of both inbred lines (set A and set B) analyzed in autumn 2010. Significant differences between the treatments are indicated by different letters (n = 3–4, *p*≤0.05, one-way ANOVA).

As shown in Figure 1A we observed obvious differences in plant growth behavior between the two sets of B73 plants with and without colonization by AM fungi. Especially under –AM conditions where plants were highly Pi-starved, plants of set A grow taller with erected leaves while set B plants stay smaller with overhanging leaves. Differences in the growth phenotype are also present under +AM condition. Here plants of set B seem to form an increase in overall leaf health, which is visible by greener, thicker and even more overhanging leaves. These growth differences were apparent in two independent experiments. To quantify these observations, dry weight, leaf number and size of the plants have been determined in autumn 2010. Here, plants from set B showed a significant difference in dry weight and number of green, non-senescent leaves in −AM versus +AM conditions (Figure 1B). In other words, under these conditions set B plants but not set A plants exhibited a strong positive mycorrhizal growth response with *G. intraradices* when access to Pi was limited to the mycorrhizal uptake pathway. Under high Pi conditions (plants have been grown at the universities of Hamburg and Regensburg in semi-sterilized soil supplemented with fertilizer) a significant difference in plant growth behavior was not observed between sets A and B (data not shown).

### Analysis of Elemental Composition

**Figure 2:**
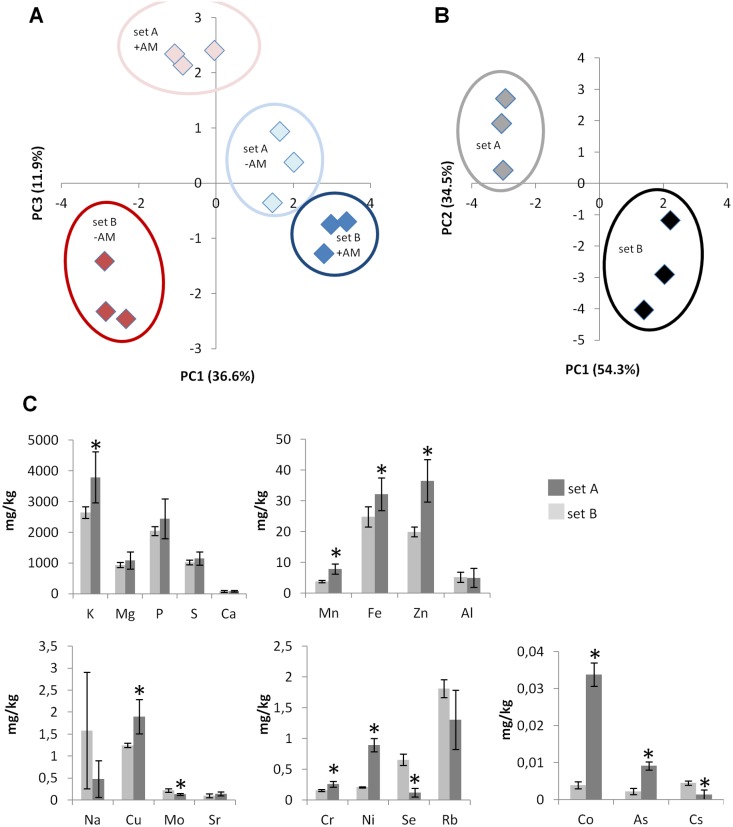
Comparison of elemental composition in source leaves and dry seeds of progeny plants from both B73 inbred lines (set A and B). (**A**) PCA-analysis of ionomics data from source leaves. Three plants were each grown in compartmented pots with (+AM) or without (−AM) arbuscular mycorrhiza with phosphate addition to the hyphal compartment. (**B**) PCA-analysis of ionomic data from dry seeds (n = 3). (C) Depiction of 20 elements separately as bar charts. Significant differences between plants of set A and set B are labeled by an asterisk (n = 4–5, student’s t-test, *p*≤0.05).

The measurement of the total elemental composition (ionomics) by inductively-coupled plasma mass spectrometry (ICP-MS) in source leaves of maize plants underlined the observed growth differences between plants of set A and B under +AM and –AM conditions (Figure 2A). A principal component analysis (PCA), which reduces all analyzed traits per treatment into few components showed a strong discrimination between –AM and +AM plants of set B (PC1 = 36.6%) while plants of set A reveal just a weak effect in PC1. In particular, P concentration is significantly increased in mycorrhizal set B plants (Figure S1). In plants of set A, a slight discrimination of +AM and –AM plants by a third PC is visible (PC3 = 11.9%). Ionomic data from the seeds used for all studies of this work showed significant differences between both sets of B73 plants. PCA analysis separated seeds of set A from seeds of set B by a PC1 of 54.3% (Figure 2B). This includes a higher accumulation of diverse elements (e.g. K, Fe, Mn, Zn, Cu, Ni, Co) in seeds of set A accompanied by a reduction of Na, Se, Cs and Mo concentrations (Figure 2C).

### Whole Genome Sequencing

**Figure 3:**
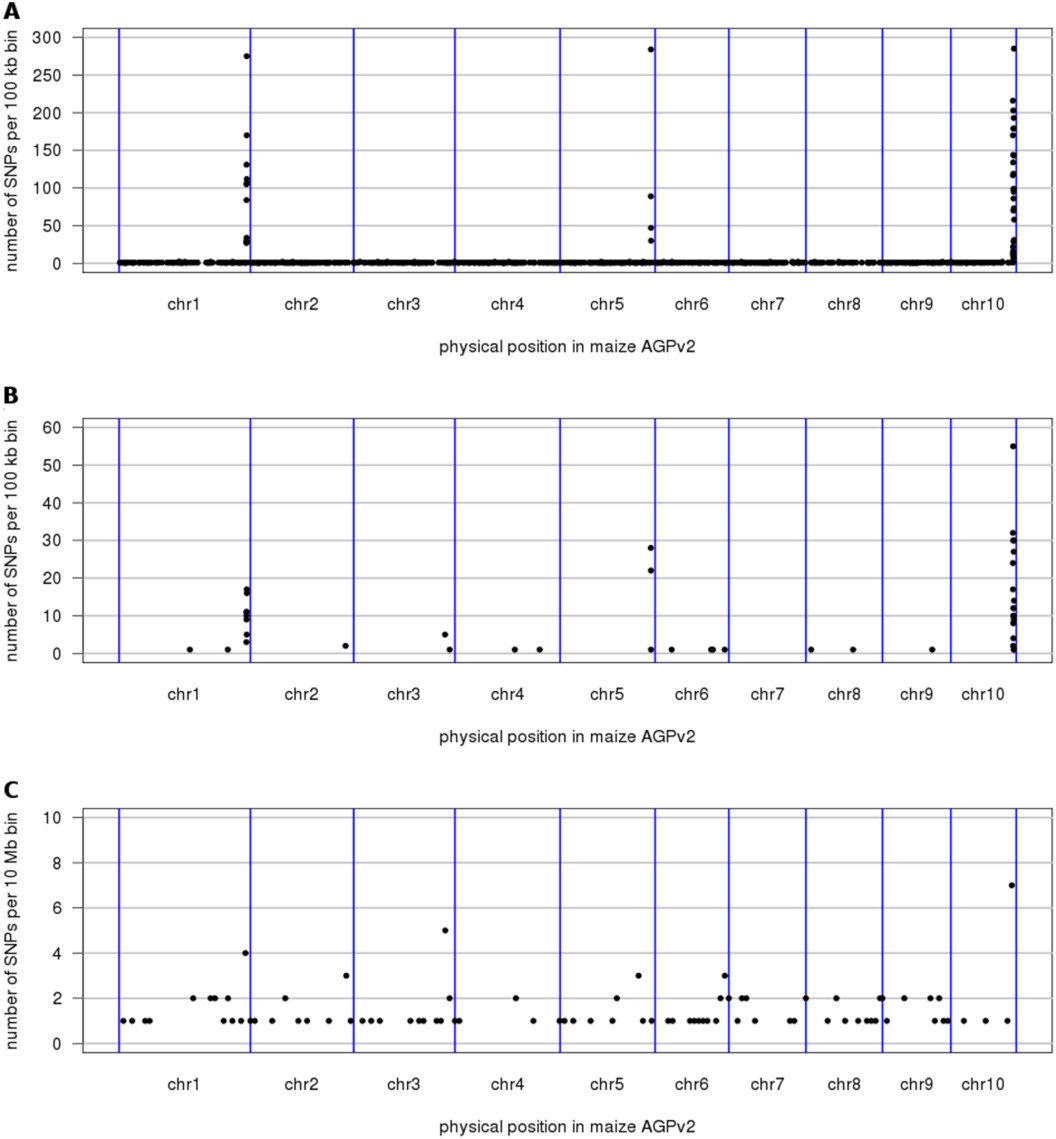
Distribution of SNPs and differentially expressed genes along the length of the 10 maize chromosomes (separated by blue lines). (**A**) Number in non-overlapping 100 kb windows of genomic SNPs differentiating between plants of set A and B, respectively. (**B**) Number in non-overlapping 100 kb windows of transcriptome SNPs differentiating between the three replicates from set A and the three replicates from set B. (C) Number in non-overlapping 10 Mb windows of differentially expressed genes (q value ≤0.05, log fold change ≥2).

**Figure 4:**
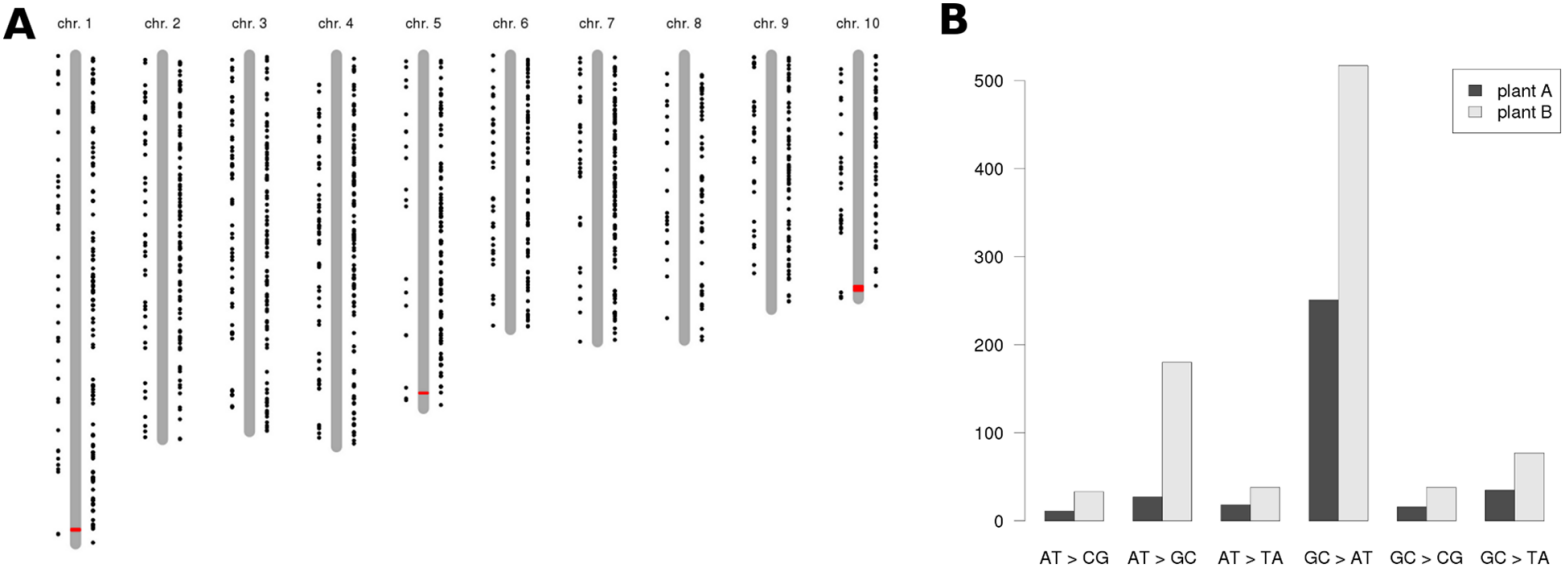
Analysis of putative *de novo* SNPs and introgression loci in both B73 inbred lines. (**A**) Distribution of putative *de novo* SNPs along the maize genome. *De novo* SNPs of set A plants are shown to the left of the chromosome ideograms, SNPs of set B plants are shown to the right. The locations of the putative introgressions on chromosomes 1, 5, and 10 in plant B are highlighted in red and drawn to scale. (**B**) Spectrum of *de novo* mutations in set A and set B plants, respectively.

As indicated above the observed differences between both sets of B73 plants may have been caused by genetic or epigenetic effects occurred during propagation of seed material for many generations either in the green house or in the field. As the genome of inbred line B73 has been sequenced and assembled to high-quality, we therefore sequenced the genome of plants from both sets. We performed whole genome shotgun (WGS) sequencing of a single plant from both set A and set B (plant A and plant B), respectively. We sequenced both plants to ∼15x whole genome coverage using the Illumina sequencing-by-synthesis platform (Table 1). Sequence reads were mapped against the maize reference sequence (AGPv2). Around 94% of all reads could be mapped. *In silico* detection of single nucleotide polymorphisms (SNPs) was performed using the SAMtools pipeline. The maize reference is characterized by a high content of repetitive elements and highly similar copies of genes as a consequence of recent allopolyploidy. Likewise, the maize reference sequence is not to be considered a finished reference sequence as the sequence has only been placed to BAC-level resolution (100–200 kb), but sequence contigs have not been ordered within single BACs. These factors caused some obstacles to accurate read mapping and variant calling from short read NGS data. We therefore focused our attention on SNPs that had sufficient (≥10x) read coverage in both samples and which were called as “homozygous alternative” in one sample and “homozygous reference” in the other. A total of 5,615 SNPs met our criteria. Of these, 358 had the non-B73 allele in plant A and 5,257 had the non-B73 allele in plant B. We calculated the number of SNPs in non-overlapping 100 kb bins. SNPs were distributed uniformly across the genome in set B (Figure 3A). While there were at most three SNPs per bin in plant A, we found 19 bins with more than 100 SNPs in plant B. The maximal SNP count per bin was 285. All bins with more than two SNPs were located in three genomic regions: Chr. 1, 292.1–293.2 Mb; Chr. 5, 208.1–208.6 Mb; Chr. 10, 142.3–145.3 Mb (Figure 3A, Figure 4A, Table 2). Of the 5,615 SNPs, 1,097 (19.5%) were located in annotated exons. Among these, 506 were predicted to have an effect on the protein sequence with four of them located on Chr. 10 introducing premature stop codons and one generating a start-codon loss (Table S1). While the latter gene (GRMZM5G874366_T01) encodes a precusor of a receptor-like protein kinase 1, two of the other candidate genes encode proteins with homology to a serine/threonine-protein phosphatase (GRMZM2G096107_P01) and a N-lysine methyltransferase-like protein (AC204437.3_FGP004).

**Table 1:** Whole genome sequence (WGS; genomic) and transcriptome (RNA) data generated in this study from B73 inbred lines of set A and B plants, respectively.

Seed set	Sample	Read type^a^	All reads	Mapped reads
A	Genomic	PE	196 M	185 M (94.6%)
	Genomic	SE	132 M	124 M (94.0%)
	RNA replicate 1	PE	145 M	123 M (85.7%)
	RNA replicate 2	PE	166 M	139 M (84.6%)
	RNA replicate 3	PE	168 M	140 M (83.3%)
B	Genomic	PE	212 M	200 M (94.0%)
	Genomic	SE	140 M	132 M (93.9%)
	RNA replicate 1	PE	130 M	108 M (82.7%)
	RNA replicate 2	PE	118 M	101 M (85.9%)
	RNA replicate 3	PE	126 M	105 M (83.1%)

a PE - paired end reads (2×100 bp), SE – single end reads (100 bp).

**Table 2:** Introgression intervals in the genome of set B plants of maize inbred line B73.

Region	No. of SNPs betweensets A and B in WGSdata^a^	No. of SNPs betweensets A and B intranscriptome data^a^
Chr1∶ 292.1–293.2 Mb	1,103 (20.9%)	46 (15.2%)
Chr5: 208.1–208.6 Mb	450 (8.5%)	28 (9.2%)
Chr10: 142.3–145.3 Mb	2,809 (53.4%)	224 (73.9%)
Complete genome	5,257 (100%)	303 (100%)

a only SNPs within the non-B73 allele in set B plants were counted.

To validate our SNP calls, we compared them against the maize HapMap version 2, which includes the genotypes of 103 lines of domesticated maize and teosinte at 55 million SNP positions. Only 24 (6.7%) of the 358 SNPs between the B73 reference and plant A were present in HapMap2. By contrast, 3,962 (70.5%) of 5,257 SNP of plant B were polymorphic in the HapMap panel. This led us to the conclusion, that plant B most likely carries an introgression from a different maize genotype in a B73 background, whereas the genome of plant A is almost identical to the B73 reference, with most SNPs attributable to errors in read mapping or SNP calling. We tried to find a possible donor genotype of the introgressed segment among the 103 HapMap lines. However, a HapMap genotype that is highly similar to plant B could not be identified.

### Transcriptome Sequencing

As we only sequenced one individual plant grown from each set A and B, respectively, we could not rule out the possibility that further introgressions are present in other plants from set B. We therefore performed transcriptome sequencing of plants grown under the same conditions from three randomly selected seeds of both sets (Table 1). RNA-seq data was used to compare SNPs called in the transcriptome data against the SNPs discovered in the genomic data and to detect consistent differences in transcript abundance between both sets. RNA was extracted from 16 d old seedlings to (i) avoid maternal effects from differentially propagated seeds and (ii) to avoid long-term environmental influences, which may alter gene expression in older partially stressed plants. Sequencing was performed using an Illumina instrument. Resulting sequence reads were mapped against the maize reference sequence. Approximately 82–86% of all reads could be mapped against the B73 reference sequence (Table 1). We called genotypes from the mapping files of each sample at the 5,257 putative variant positions discovered by whole genome sequencing. We required that at least 10 RNA-seq reads be present to make a genotype call. A total of 567 SNPs were successfully typed in the three samples from set A. Genotype calls were in complete agreement with the calls made from the WGS shotgun data. In five cases, plants from set A had the non-B73 allele. In the plant from set B, genotypes for 309 SNPs were called in all three replicates. Genotypes of all but one SNP agreed with the WGS data. The single discordant SNP was called heterozygous in the RNA-seq data, while it was called homozygous non-B73 in the WGS data. Among the other SNPs, 303 (98%) had the non-B73 allele and five had the B73 allele (Table 2).

We also determined the number of SNPs called across all six samples and differentiating between set A and B (i.e. all plants from set A are homozygous for one allele and all plants from set B are homozygous for the other allele) without making use of SNP positions discovered in the WGS data. Visualization of the number of these SNPs in 100 kb bins identified the same intervals of increased SNP density (Figure 3B). The agreement between SNPs detected in the WGS and in the RNA-seq data supports the hypothesis that all set B plants harbor the same non-B73 segment. To exclude the possibility of other unnoticed non-B73 segments in either set, we plotted the counts of heterozygous and homozygous SNPs (relative to the B73 reference) in the eight sequenced individuals (Figures S2 and S3). No other intervals with an increased number of homozygous SNPs were discovered. The number of heterozygous calls was higher in all samples compared to the number of homozygous calls. Several isolated 100 kb bins showed an elevated number of heterozygous SNPs. Three bins (Chr3∶ 186.6 Mb, Chr7∶ 162.2 Mb and Chr9∶ 7.5 Mb) had more than 20 heterozygous SNPs in at least one RNA-seq sample and at least 14 SNPs in all six RNA-seq samples. These intervals are most likely paralogous regions that are collapsed in the B73 genome assembly. Two other bins (Chr1∶ 293.0 Mb and Chr10∶ 142.4 Mb) within the putative introgression intervals (Table 2) had at least 17 SNPs in all three replicates from set B and less than two SNPs in the samples from set A. These intervals could be the results of small regions of residual heterozygosity or paralogous SNPs in tandem duplicated regions that occur in single copy number in B73, but in higher copy number in the unknown donor of the introgression.

We used the RNA-seq data collected from the six samples to analyze differential transcript abundance between plants from set A and set B. Overall 121 genes (Table 3, Table S2) were found to be differentially expressed (DE) at a liberal threshold (corrected p-value ≤0.05 and log fold change ≥2). DE genes were also present outside of the introgression intervals on chromosome 1, 5, and 10 (Figure 3B). Only nine out of 121 DE genes were located in the introgression intervals (Table S2). This is not unexpected as sequence polymorphisms within the introgressed segments may alter the abundance of transcripts or their encoded proteins and these may function subsequently as *trans-*acting factors influencing the expression of transcripts from loci in other genomic regions. Transcripts >10 times up-regulated in set A compared with set B include three genes involved in the anthocyanin biosynthesis pathway: the Anthocyanin regulatory C1 protein (GRMZM2G005066), a Dihydroflavonol-4-reductase (GRMZM2G026930) and an Anthocyanidin 3-O-glucosyltransferase (GRMZM2G165390). Activation of the anthocyanin pathway is a well-known reaction of plants to Pi stress [22] and may be related to the different growth phenotype observed between set A and set B plants. Other strongly up-regulated genes in set A are involved in plant hormone metabolism such as auxin conjugation (Indole-3-acetate beta-glucosyltransferase; GRMZM2G078465) and generation of active cytokinin (Cis-zeatin O-glucosyltransferase; GRMZM2G110511) as well as hormonal responses (SAUR14 - auxin-responsive SAUR family member; GRMZM2G447151) that may explain the AM independent differences in growth behavior. Genes strongly up-regulated in set B are involved, for example, in pyruvate metabolism (Isochorismatase family protein rutB), gene regulation (Homeobox-leucine zipper protein with homology to ATHB-4; GRMZM2G126239) and RNA metabolism (Splicing factor U2af 38 kDa subunit; GRMZM2G031827). The NIL identified in this study thus additionally provides an elegant tool to study the genetic and biochemical effects of genes that are differentially expressed between both sets of B73 inbred lines.

**Table 3:** Top20^a^ differentially expressed genes between B73 inbred lines of set A and B with functional annotation.

Gene	Locus	FPKM_A	FPKM_B	Functional annotation^b^
GRMZM2G049021	5∶133618125–133626411	0.12	54.09	Isochorismatase familyprotein rutB
GRMZM2G078465	7∶140007358–140009147	51.34	1.32	Indole-3-acetate beta-glucosyltransferase
GRMZM2G026930	3∶216304733–216306568	2.21	0.1	Dihydroflavonol-4-reductase
GRMZM2G031827	8∶70136196–70144793	0.77	15.21	Splicing factor U2af 38 kDasubunit
GRMZM2G110511	8∶167407655–167409403	1.06	0.08	Cis-zeatin O-glucosyltransferase 2(cisZOG2)
GRMZM2G126239	3∶214857326–214861719	0.47	5.52	Homeobox-leucine zipper proteinATHB-4
GRMZM2G016890	10∶34232716–34238135	0.41	4.25	Beta-glucosidase,chloroplastic
GRMZM2G000620	10∶144143810–144147098	11.18	1.07	Receptor-like kinase
GRMZM2G165390	9∶11774732–11776491	1.62	0.16	Anthocyanidin 3-O-glucosyltransferase
GRMZM2G161905	9∶155596297–155597233	11.15	1.13	Glutathione S-transferaseGST 25 Fragment
GRMZM2G005066	9∶9740802–9741876	6.13	0.64	Anthocyanin regulatoryC1 protein
GRMZM2G073916	8∶91681549–91689395	0.12	1.13	Protein aq_1857
GRMZM2G047368	7∶41539129–41540625	147.58	17.41	Aquaporin PIP2–6
GRMZM2G447151	1∶258800054–258800675	25.56	3.29	SAUR14 - auxin-responsive SAURfamily member
GRMZM2G179294	5∶180731982–180733210	0.45	3.12	High affinity nitratetransporter
GRMZM2G035444	1∶172897702–172899238	0.21	1.26	UPF0497 membraneprotein 3
GRMZM2G151227	2∶223888705–223892691	15.35	2.58	Chalcone synthaseWHP1
GRMZM2G063244	8∶1141429–1145642	1.57	0.27	Peptidyl-prolyl cis-trans isomerase
GRMZM2G046952	10∶132098076–132099489	0.29	1.54	FIP1
GRMZM2G025459	8∶120217908–120221032	0.52	2.79	SNF1-related protein kinaseregulatory subunit beta-1

a a full list of all differentially expressed genes is available as Table S2.

b only genes with functional annotation are shown.

### De Novo Mutations

Resequencing studies in several model organisms, such as *Arabidopsis thaliana* [23], yeast [24] and *Caenorhabditis elegans* [25], have revealed that genomes accumulate spontaneous mutations at a rate of the order of 10^−9^ to 10^−8^ per site per generation. We mined our genomic variants calls for SNPs to the B73 reference sequence that occur either in set A or set B plants (but not in both) and are not part of HapMap2, and considered these “*de novo* SNPs” to be the result of spontaneous mutations that occurred during propagation of the seed stock. A total of 358 and 883 *de novo* SNPs were found in the genome of set A and set B plants, respectively. These putative *de novo* mutations were located approximately uniformly along the ten maize chromosomes (Figure 4A). Taking the mutation rate in *A. thaliana* (7×10^−9^ base substitutions per site per generation) [23] as a proxy for the mutation rate in maize, ∼16 mutations occur in the 2.3 Gbp genome of maize per generation, indicating that the B73 reference is ∼22 generations removed from the genomes of set A plants and ∼55 generations from the genome of set B plants. Note that these numbers count propagation cycles starting from a putative common ancestor of both the reference genome and set A/B pkants. The spectrum of *de novo* mutations was largely similar between set A and B plants (Figure 4B). The majority of mutations were G:C -> A:T transitions, similar to findings in *A. thaliana* [26]. Mutations were distributed more or less uniformly along the chromosomes. Spontaneous mutations in protein-coding genes may result in differential gene expression and could be a possible cause for the phenotypic differences of set A and B plants. Seven of the putative *de novo* SNPs of set A plants and 14 of those of set B plants are located in annotated exons. Out of these exonic SNPs, only one was found within a differentially expressed gene (GRMZM2G132956). The difference in transcript abundance of this gene was statistically significant (p = 0.003), but the log2 fold change was only 0.77. Given the absence of a functional annotation, we did not consider this gene as a good candidate for a causal gene underlying the described growth and ionome phenotypes.

## Discussion

We have described here a nearly isogenic line (NIL) of maize inbred line B73. The NIL was found by chance when we compared the response of field and green house grown plants to Pi starvation and AM fungi in a wider panel of maize inbred lines. Phenotypic changes were accompanied by alterations in transcript levels and the ionomic composition of leaves and seeds. Through whole-genome and transcriptome sequencing, we could ascertain that the NIL is pure and harbors three small introgression intervals on chromosome 1, 5 and 10 in a B73 genomic background. The analysis of differential gene expression revealed consistent differences in transcript abundance between both sets of plants.

We were not able to trace back the origin of this NIL. It could be speculated that the introgression was introduced by unintentional and unnoticed cross-fertilization with an unknown parent during seed propagation at the maize stock center. Several rounds of backcrossing to “genuine” B73 may have decreased the size of the genomic regions inherited by the non-B73 parent. Subsequent rounds of self-fertilization may subsequently have led to fixation of the non-B73 allele in a homozygous state in three introgression intervals. This theory requires that in the “development” of the NIL backcrossing by unsupervised pollination in a plot of supposedly homogeneous B73 plants was supplanted with artificial self-pollination. Another possible explanation would be that B73 is not an entirely homogeneous inbred line, but to some extent a “fluid concept”: B73 lines maintained at different locations (or at the same location at different time points) exhibit slight differences in sequence composition that result only in minor phenotypic alterations, which in most cases escape notice. It is thus likely that more B73 NILs are around and are used by various labs and plant breeders.

Whatever the exact crossing scheme may be, the NIL seems now to be maintained as an (almost) pure (i.e. (nearly) homozygous) line at the University of Regensburg as plants grown from different B73 seed stocks showed an altered growth phenotype under low Pi conditions and between +AM and –AM treatment. Likewise, sequencing of RNA samples of three B73 plants randomly selected at Regensburg (set B plants) revealed the same three genomic intervals in all three samples and in the plant sequenced by whole-genome shotgun sequencing. As only controlled self-pollination has been carried out in the greenhouse and the number of SNPs is very small, it is unlikely that cross-pollination occurred at the Universities of Hamburg and Regensburg after seeds had been obtained from the stock center. The combined size of all three introgression intervals is about 4.6 Mb, i.e. ∼0.2% of the entire maize genome. The introgressed segments are located in highly recombinogenic subtelomeric regions of the long arms of the respective chromosomes. The recombination rate at these regions is approximately 3 cM per Mb [27]. The genetic length of the introgression interval is approximately 14 cM (0.6–0.8% of the genetic length of the maize genome [27]). An initial hybrid has to be backcrossed to B73 for seven generations and subsequently selfed for several generations to decrease the introgressions size to this tiny proportion of the genome and to obtain homozygous lines. If this process is carried out unintentionally, it would most likely not result in homogeneous material. Thus, it is likely that seeds carrying the undesired introgression had already been shipped by the stock center.

This finding raises some concerns about the homogeneity of material from different sources that is supposed to have the same genetic background. Cryptic introgressions in the genomes of presumably identical individuals may result in phenotypic differences that become evident only under very special growth conditions. If slightly divergent material is inadvertently used in line development (e.g. in map-based cloning projects), mutant phenotypes may be obfuscated or, in the worst case, even emulated by variation in diverging chromosomal regions. In view of this worrying perspective, we advise that the identity of plant material, in particular when originating from different laboratories, should always be double-checked. Other reports corroborate our concerns about the purity of seed material. A small segment (1.5 Mb) that differs between different B73 lines has been reported previously [1]. Moreover, the maize haplotype map has revealed that many inbred lines are not completely homozygous, but retain considerable heterozygosity in rarely recombining pericentromeric regions [1]. Genotyping-by-sequencing (GBS) of the US maize seed bank [5] revealed residual heterozygogity as a common source of intra-accession variability. Next generation sequencing in conjunction with cost-effective means of genomic complexity reduction [28], [29] has enabled the simultaneous interrogation of thousands of molecular markers in a large number of individuals. In the future, it may become possible to monitor the genomic identity of each individuals involved in a specific experiment as well as to validate the identity and homozygosity of samples distributed by genebanks [5].

We are fully aware that the data reported in this study does not constitute a definite proof that a gene or sequence variant underlying the different growth phenotype is located in the three introgressions intervals delineated by high-throughput sequencing. We have found several hundred putative *de novo* SNPs of the two lines relative to the B73 reference genome. Only one of these was located in the coding exon of a differentially expressed gene and this gene was not an obvious causal candidate underlying the growth phenotype. It is possible that, for example, a complex structural rearrangement such as a transposon insertion into the regulatory sequence of a gene residing outside the putative introgressions has affected the expression of one or several genes, which subsequently brought about the differential growth response to Pi starvation in the presence or absence of AM fungi. We can also not rule out epigenetic differences between both lines, though these have to be stably inherited as we saw consistent differences between all plants of the two B73 lines.

We moreover cannot exclude that overall growth responses could be influenced by the different initial nutrient content in the two seed batches (Figure 2 B, C). This may affect seedling’s growth in the first weeks after germination on low nutrient substrate as shown for dicotyledonous species [30]. Under our experimental conditions non Pi-fertilized plants suffer from Pi-limitation approximately two weeks after germination. Latest at this time point internal nutritional reserves should be completely depleted. This was also one reason why we have chosen seedlings at 16 days after sowing for transcriptomic studies. Interestingly higher nutrient concentration of set A seeds for many growth-relevant elements like K, Mn, Zn and Fe disagrees with a finally higher biomass accumulation of six week old set B plants. Moreover no additional differences in P concentration of both seed batches (Figure 2 C) but significantly higher P-concentrations in mycorrhizal source leaves of set B (Figure S1) were found. This altogether points against maternal effects by the different nutrient content of the seeds and to a direct influence of the altered genetic background of set B plants towards improved mycorrhizal responsiveness including altered nutrient uptake. Particularly the mycorrhizal uptake pathway of set B plants could be induced by alterations in the mycorrhizal signal transduction cascade, which may result into an increased uptake of essential nutrients (e. g. P) and therefore improved growth. This hypothesis is supported by the finding that genes encoding a novel leucine-rich repeat receptor-like protein kinase family protein as well as a SNF1 protein kinase regulatory subunit are strongly induced in set B plants (see also Table 3 and Suppl. Table S2). Further analysis of gene expression in roots of both B73 maize lines may highlight putative differential responses of nutrient transporter genes like the mycorrhiza-specific phosphate transporter Pht1; 6 [12] or ammonium and nitrate transporters [31]. The improved ability of nutrient transport within set B plants like nitrogen-transport is reflected, for example, by the increased expression of a high affinity nitrate transporter (GRMZM2G179294) (Table 3).

As gene expression analysis has been conducted with young seedlings under fully fertilized conditions without AM fungi, thus a direct link between increased mycorrhizal responsiveness and the differential gene expression results requires further experimentation. Nevertheless it is conspicuous that differential gene regulation between set A and B plants points to an overall elevated stress response level of set A plants. In particular the strongly increased expression of genes involved in the flavonoid and especially anthocyanin biosynthesis pathway (such as Dihydroflavonol-4-reductase, Anthocyanidin 3-O-glucosyltransferase, Chalcone synthase WHP1, Phenylalanine ammonia-lyase and the major transcriptional regulator of the pathway, C1 [32], see also Table 3 and Suppl. Table S2) might not only mirror higher Pi-stress of set A plants even under full fertilizer conditions, but additionally indicate higher vitality of these plants to general stress conditions. Under Pi-limited conditions these plants also visually look more Pi-starved with erected leaves and an overall non-compact growth phenotype compared with set B plants (Figure 1). Moreover expression of the heat shock protein genes HSP26 and HSP101 is increased in set A seedlings, which is typically expressed at high levels under stress reactions [33] as well as the strongly increased expression of a Glutathione S-transferase gene, which is required to detoxify endogenous compounds and xenobiotics such as herbicides [34].

In summary the strong up-regulation of flavonoid biosynthesis and stress related genes in set A plants suggest a higher fitness of these plants, while an increase of kinase genes might be associated with signaling for mycorrhizal uptake pathways in set B plants. These hypothesis require further experimentation, but the NIL described in the present study can now form the basis for further research to fine-map, for example, the loci responsible for the altered growth phenotype of set B plants. Moreover, genetic mapping could be employed to confirm or reject the association of genomic introgression intervals and the phenotype. For example, plants of an F2 population obtained by crossing the AM line with B73 could be scored for their growth phenotype and genotyped with cost-efficient GBS technology [29]. As all three introgression intervals are located in distal, highly recombinogenic regions, mapping in even a small F2 population of 50–100 plants may delimit a reasonably small target region, which can then be mined for candidate genes with the assistance of the genomic and transcriptomic data reported in this study. Finally, the NIL reported in this study provides a valuable resource for functional studies of above described genes differentially expressed between both sets of plants such as the genes involved in flavonoid biosynthesis.

## Materials and Methods

### Plant Material and Growth Conditions Prior to DNA/RNA Extraction for Sequencing

B73 maize lines were obtained from the USDA stock center in the early 1990s (named as set B in this study) and in 2007 (Acc.-No.: PI 550473; named as set A). Both sets were propagated exclusively after self-pollination either in the field (set A) or in the greenhouse (set B). Seed material of set B plants is available by request to the corresponding author. Plants were grown in the greenhouse with supplemental light under long-day conditions (16 h light/8 h dark) at 27°C during the day and 18.5°C during the night.

### Genomic DNA Preparation for Illumina Sequencing

Leaf material from 12 day old plants at stage V3 were sampled and immediately frozen in liquid nitrogen. One gram of leaf tissue each was ground in a mortar under liquid nitrogen and genomic DNA was prepared using the DNeasy Plant Maxi Kit (Qiagen) according to the instructions of the manufacturer.

### RNA Preparation for Illumina Sequencing

Material of the third leaf from sixteen day old plants at stage V4 were sampled and immediately frozen in liquid nitrogen. Hundred mg frozen leaf tissue each was ground in a swing mill and RNA was prepared using the RNeasy Mini Kit (Qiagen) according to the instructions of the manufacturer.

### Sample Preparation and Illumina Sequencing

Genomic DNA was fragmented to a target size of 200 bp using the Covaris S2 and the DNA microTUBE protocol (KBiosciences). Indexed libraries were prepared with the TruSeq DNA Sample Preparation Kit (Illumina) and were subsequently quantified with the KAPA Library Quantification Kit for Illumina (KAPA Biosystems) and the DNA 1000 assay on the 2100 Bioanalyzer (Agilent).

*Single end reads*: Cluster generation on the cBot was performed using the TruSeq SR Cluster Kit v2, followed by a 101 cycles standard single-read sequencing run on the HiScanSQ using the TruSeq SBS Kit v2. *Paired end reads*: Cluster generation on the cBot was performed using the TruSeq PE Cluster Kit v3, followed by a 2×101+7 cycles multiplexed paired-end run on the HiSeq 2000 instrument using the TruSeq SBS Kit v3.

RNAseq and preparation of the respective libraries were carried out as described in the Illumina TruSeq RNA Sample Preparation Guide, the Illumina HiScan 1000 System User Guide (Illumina), and the KAPA Library Quantification Kit - Illumina/ABI Prism User Guide (Kapa Biosystems). In brief, 1 µg of total RNA was used for purifying the poly-A containing mRNA molecules using poly-T oligo-attached magnetic beads. Following purification, mRNA was fragmented to an average insert size of 250–450 bases using divalent cations under elevated temperature (94°C for 4 minutes). Cleaved RNA fragments were copied into first strand cDNA using reverse transcriptase and random primers followed by second strand cDNA synthesis using DNA Polymerase I and RNase H. The resulting cDNA fragments subsequently went through an end repair process, the addition of a single ‘A’ base, the ligation of the adapters, and a purification step. Finally cDNA libraries were created by PCR enrichment. Libraries were quantified using the KAPA SYBR FAST ABI Prism Library Quantification Kit. Equimolar amounts of each library were used for cluster generation using the cBot (TruSeq PE Cluster Kit v3). Sequencing runs were performed on a HiSeq 1000 instrument using the indexed, 2×100 cycles paired end (PE) protocol and the TruSeq SBS v3 Kit. Image analysis and base calling resulted in.bcl files, which were converted into.fastq files by the CASAVA1.8.2 software.

Genomic DNA and RNA fragmentation, library generation and sequencing were performed at the local genomics core facility “KFB - Center of Excellence for Fluorescent Bioanalytics” at the University of Regensburg. Sequence data have been deposited under the SRA accession numbers PRJEB4837 (WGS data) and PRJEB4838 (RNA-seq data).

### Bicompartmented System

Maize plants were grown in a bicompartmented system consisting of a root hyphal compartment (RHC) and a hyphal compartment (HC) containing 80 mg KH_2_PO_4_. By this experimental approach, Pi could be taken up by the mycorrhizal phosphate uptake pathway. Individual maize plants were grown in 1.1 kg of a sand:soil (0.71–1.25 mm; Quarzwerke GmbH)/soil (Stender Vermehrungssubstrat A210, Stender) mixture (9∶1) in a rectangular container (root and hyphal compartment, RHC). The substrate was supplemented with roots of *Plantago lanceolata* colonized by *Glomus intraradices* Schenck and Smith (BEG75) (syn: *Rhizophagus irregulare*) (72 g/kg substrate) for the +AM treatment. As a hyphal compartment a small plastic vial was added upside down to the container including 40 g of a sand/soil mixture without inoculum and 80 mg of KH_2_PO_4_ as a Pi source. The plastic vial separates maize plant and Pi source via a semipermeable membrane, which is only permeable by fungal hyphae but not by roots of the plant. Three times a week plants were fertilized with a modified single strength Hoagland’s nutrient solution without Pi (NH_4_PO_4_ replaced by NH_4_Cl). Water-holding capacity was maintained at 75%. Plants were grown in the greenhouse at 24°C/20°C day/night temperature for 6–8 weeks. The first completely developed leaf (first source leaf) was harvested for ionomic analysis. For each condition/genotype three to four replicates were analyzed. For determination of the colonization degree by trypan blue staining a representative root sample was harvested per plant [35]. Above- and belowground plant material was dried to stable weight for biomass determination. Leaf number and the number of green leaves was determined.

### Ionomics Analysis

A microwave system (Multiwave 3000, Anton Paar) was employed for complete digestion of plant material for ICP-MS analysis. Approximately 0.1 g of dried and homogenized plant material (leaves, seeds) was digested using 4 ml of concentrated HNO_3_ (66%) and 2 ml of H_2_O_2_ (33%). The microwave run started with a 10 min power ramp followed by 30 min at 1400 W and finished with 15 min of cool down. A certified reference material “hay powder” (Community Bureau of Reference, No 129) was used for control of the digestion quality. A 7700 ICP-MS instrument (Agilent) was employed for determination of approx. 20 elements following the manufacturer’s instructions.

### Read Mapping and Variant Calling

WGS reads were mapped against the maize reference sequence (AGPv2) with BWA [36] version 0.5.9. The BWA command “aln” was called with the parameters “-I –q 20” for trimming off bad-quality at the ends of reads. Default BWA parameters were used otherwise. Variant and genotype calling were performed with SAMtools [37] version 0.1.19. The command “samtools mpileup” was called with the parameter “-D” to record per-sample read depth. The resulting VCF file was filtered with an AWK script (available as Text S3 of [38]). SNP position were retained if they were both samples had at least 10-fold read coverage, the SAMtools SNP quality score was at least 40, both samples were called homozygous (reference or alternative allele) with minimum genotype score of 10 and both samples had different genotype calls. Maize HapMap2 [2] genotype calls were downloaded from Panzea [39]. HapMap position were intersected with our SNP set using Tabix [40]. Aggregation of variants in 100 kb bins and visualization were performed with R scripts (http://www.r-project.org). R source code is provided as Text S1. Functional annotation of variant sites was performed with snpEff (version 3.2a) [41] using the filtered gene set of maize (version 5b.60, http://www.maizesequence.org).

RNA-seq reads from six samples were mapped against the maize B73 reference sequence with the Tophat spliced-alignment program [42] version 2.0.4 (parameters “-mate-std-dev 90 -r 0”). Fragment size statistics were determined by mapping reads with BWA against maize cDNA sequences downloaded from Phytozome [8], [43]. Genotypes in all samples were called at the SNP positions determined from genomic reads. A read pileup at variant positions was generated with “samtools mpileup” for each sample. Variant positions were supplied with the parameter “-l”. The resulting VCF files were imported into the R statistical environment. A genotype call was set to missing if the read depth was less than 10.

A multi-sample calling was performed with the SAMtools pipeline from RNA-seq reads using the same parameters as described for the WGS data. For the visualization of all homozygous and heterozygous SNPs called in the genomic and RNA-seq samples, SNPs were filtered for minimal coverage (≥10x), SNP quality score (≥40) and genotype score (≥10). Filtered variant call files are available as Datasets S1 (WGS data) and Dataset S2 (RNA-seq data).

### Analysis of Differential Gene Expression

Cufflinks [44] version 2.1.1 was used for the analysis of differential gene expression. The command “cuffdiff” was supplied with the maize reference annotation [8] (filtered gene set 5b60) and the Tophat mapping file of all six RNA-seq samples divided into two contrasting groups (set A and set B). In addition, the parameters “-u” and “-b” were used for multi-read correction and fragment bias correction, respectively. Tables containing the expression values and test results for each reference gene were imported into the R environment. The number of genes that were differentially expressed at a significance threshold of 0.05 after Benjamini-Hochberg correction with a minimal log-fold change ≥2 were counted in 10 Mb windows and visualized along the length of the maize genome. Predictions of molecular functions were downloaded from Gramene [45] and merged with the list of DE genes.

## Supporting Information

Figure S1 **Determination of P-concentration in source leaves of both plant sets by ICP-MS analysis.**
(TIF)

Figure S2 **Number of homozygous SNPs in 100 kb bins for all sequenced samples (2x WGS, 6x RNA-seq).**
(TIF)

Figure S3 **Number of heterozygous SNPs in 100 kb bins for all sequenced samples (2x WGS, 6x RNA-seq).**
(TIF)

Table S1 **Functional annotation of 5,615 SNP variant sites.**
(XLS)

Table S2 **List of 121 differentially expressed genes with functional annotation.**
(XLSX)

Text S1 **R source code used to generate SNP frequency plots.**
(TXT)

Dataset S1 **Filtered SNPs in the WGS data.**
(TSV)

Dataset S2 **Filtered SNPs in the RNA-seq data.**
(TSV)
