# Supplementary figures and images for: Sequence and Ionomic Analysis of Divergent Strains of Maize Inbred Line B73 with an Altered Growth Phenotype
Source: PLoS One. 2014 May 7;9(5):e96782. doi: 10.1371/journal.pone.0096782 (PMC4013074; doi:10.1371/journal.pone.0096782)

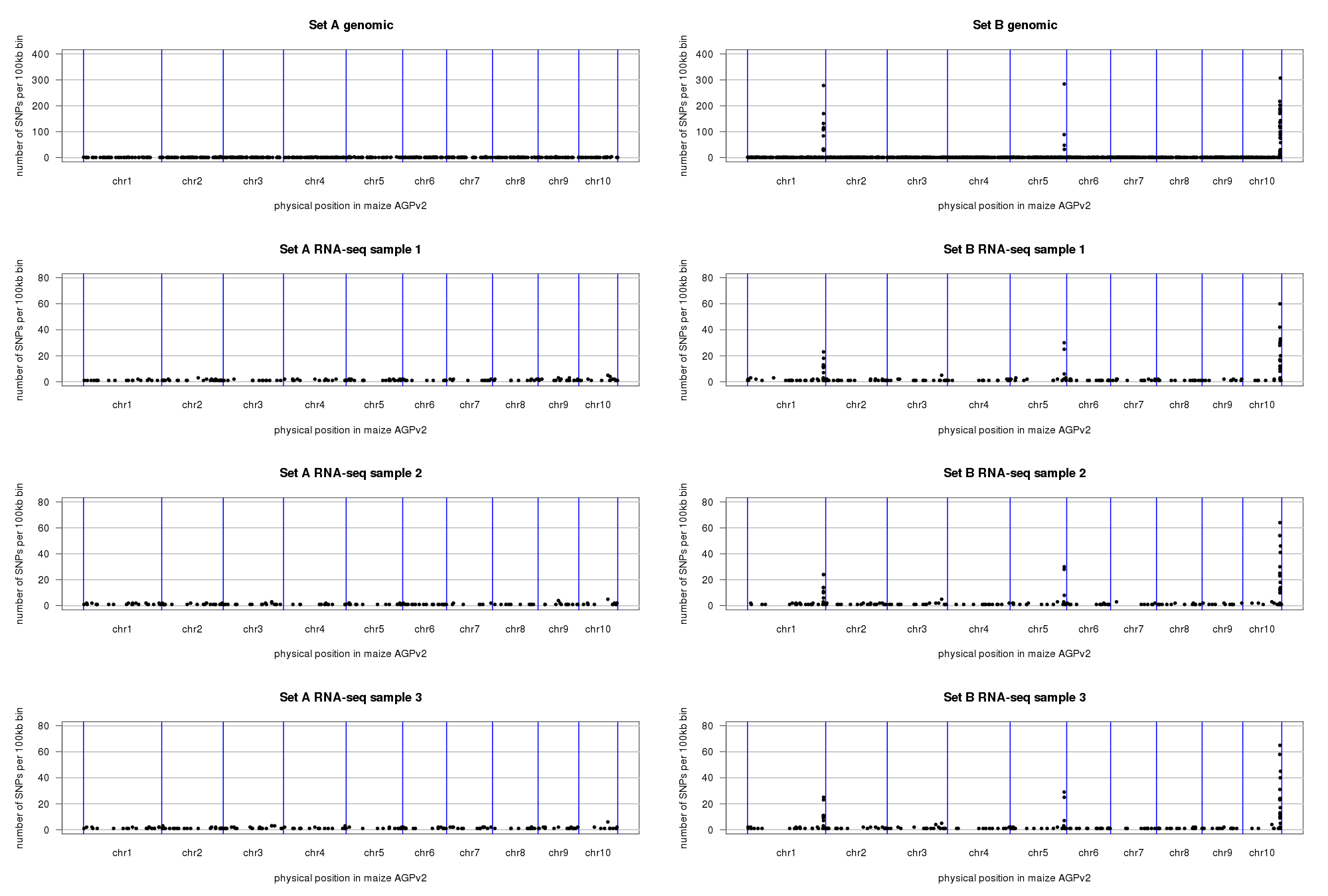

Supplement: Figure S2 — Number of homozygous SNPs in 100 kb bins for all sequenced samples (2x WGS, 6x RNA-seq). (TIF) [file pone.0096782.s002.tif]

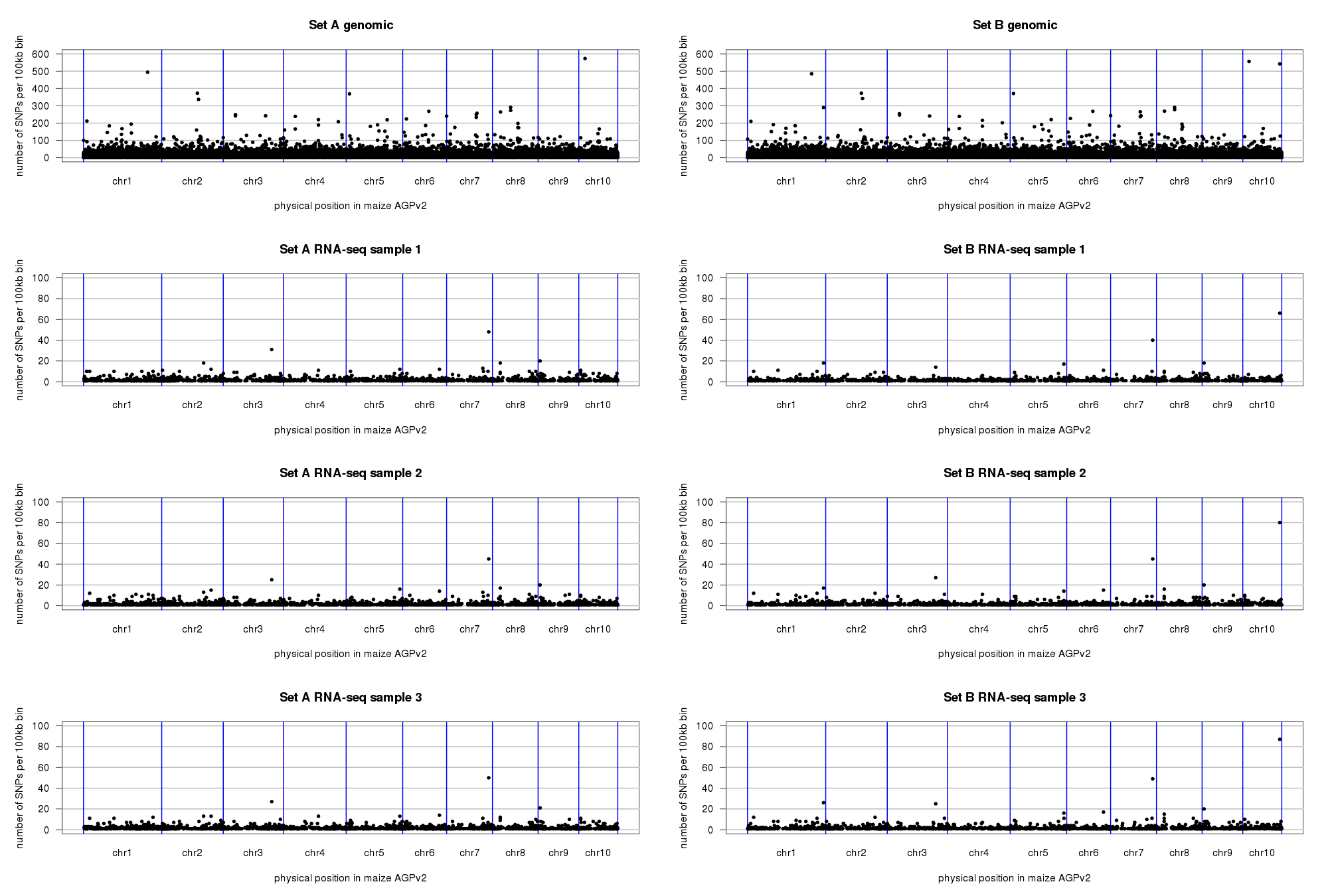

Supplement: Figure S3 — Number of heterozygous SNPs in 100 kb bins for all sequenced samples (2x WGS, 6x RNA-seq). (TIF) [file pone.0096782.s003.tif]
